# Hesperidin Protects against Acute Alcoholic Injury through Improving Lipid Metabolism and Cell Damage in Zebrafish Larvae

**DOI:** 10.1155/2017/7282653

**Published:** 2017-05-17

**Authors:** Zhenting Zhou, Weichao Zhong, Haiyan Lin, Peng Huang, Ning Ma, Yuqing Zhang, Chuying Zhou, Yuling Lai, Shaohui Huang, Shiying Huang, Lei Gao, Zhiping Lv

**Affiliations:** ^1^School of Traditional Chinese Medicine, Southern Medical University, Guangzhou, Guangdong, China; ^2^Department of Liver Diseases, Shenzhen Traditional Chinese Medicine Hospital, Shenzhen, Guangdong, China; ^3^Key Laboratory of Zebrafish Modeling and Drug Screening for Human Diseases of Guangdong Higher Education Institutes, Department of Developmental Biology, Institute of Genetic Engineering, School of Basic Medical Sciences, Southern Medical University, Guangzhou, Guangdong, China; ^4^The Key Laboratory of Molecular Biology, State Administration of Traditional Chinese Medicine, School of Traditional Chinese Medicine, Southern Medical University, Guangzhou, Guangdong, China

## Abstract

Alcoholic liver disease (ALD) is a series of abnormalities of liver function, including alcoholic steatosis, steatohepatitis, and cirrhosis. Hesperidin, the major constituent of flavanone in grapefruit, is proved to play a role in antioxidation, anti-inflammation, and reducing multiple organs damage in various animal experiments. However, the underlying mechanism of resistance to alcoholic liver injury is still unclear. Thus, we aimed to investigate the protective effects of hesperidin against ALD and its molecular mechanism in this study. We established an ALD zebrafish larvae model induced by 350 mM ethanol for 32 hours, using wild-type and transgenic line with liver-specific eGFP expression* Tg (lfabp10α:eGFP)* zebrafish larvae (4 dpf). The results revealed that hesperidin dramatically reduced the hepatic morphological damage and the expressions of alcohol and lipid metabolism related genes, including* cyp2y3*,* cyp3a65*,* hmgcra*,* hmgcrb*,* fasn, and fads2 *compared with ALD model. Moreover, the findings demonstrated that hesperidin alleviated hepatic damage as well, which is reflected by the expressions of endoplasmic reticulum stress and DNA damage related genes (*chop*,* gadd45αa,* and* edem1*). In conclusion, this study revealed that hesperidin can inhibit alcoholic damage to liver of zebrafish larvae by reducing endoplasmic reticulum stress and DNA damage, regulating alcohol and lipid metabolism.

## 1. Introduction

Hepatic steatosis is the early stage of alcoholic liver disease (ALD) induced by alcoholic consumption. ALD is an important component of liver diseases [1]. ALD involves the processes of hepatic pathological states, from simple hepatic steatosis to progressive fibrosis, cirrhosis, and even liver cancer [2]. Given that the prevalence of ALD worldwide is rising these years, exploring an effective treatment is of great importance.

Hesperidin, a kind of citrus bioflavonoid and abundant in citrus plants, including grapefruits, oranges, and lemons, is proved to play a role in antioxidation, anti-inflammation, and cardiovascular protection [3]. In addition, hesperidin regulates hepatic cholesterol synthesis by inhibiting the activity of 3-hydroxy-3-methyl-glutaryl-CoA (HMG-CoA) reductase [4, 5]. Recently, it is confirmed that hesperidin protects against fatty liver induced by high-cholesterol diet through mediating the mRNA expressions of* rbp*,* c-fabp*, and* h-fabp*, inhibiting synthesis and absorption of cholesterol [6]. Hesperidin is also capable of attenuating liver fibrosis by mitigating oxidative stress and modulating proinflammatory and profibrotic signals [7]. However, the effects of hesperidin on alcohol-induced hepatic steatosis need further investigation and its underlying mechanisms remain unknown.

Taking into consideration findings mentioned above, we investigated the protective role of hesperidin in alcohol-induced liver injury of zebrafish larvae in the present study. We revealed the underlying mechanism of hesperidin against dyslipidemia and hepatocytes damage in ALD by evaluating the expression of some key genes related to alcohol and lipid metabolism. Furthermore, morphological observation of the whole bodies and livers of zebrafish larvae also showed the protective role of hesperidin in pathological changes caused by alcohol. First, we investigated the regulation of hesperidin on both alcohol metabolism and lipid homeostasis in zebrafish larvae ALD model and further drew the conclusion that hesperidin could resist to alcohol-induced metabolic abnormalities. Collectively, the results proved the abilities of hesperidin to reduce lipid accumulation and further demonstrated it could improve alcohol and lipid metabolism as well as hepatic steatosis. In a word, we hypothesize that citrus flavonoids are an effective treatment of ALD-related metabolic pathways through the ability of regulation of hesperidin on alcohol metabolism, lipid homeostasis, and liver damage.

## 2. Material and Methods

### 2.1. Animal Care and Treatment

Wild-type (WT) AB strain zebrafish and* Tg (lfabp10α:eGFP)* transgenics, obtained from Key Laboratory of Zebrafish Modeling and Drug Screening for Human Diseases of Guangdong Higher Education Institutes, Southern Medical University and School of Life Science, Southwest University, respectively, were cultured on a 14 h light/10 h dark cycle at 28°C following established protocols* (Westerfield M 2000 The Zebrafish Book: A Guide for the Laboratory Use of Zebrafish (Danio rerio). Eugene: Univ. of Oregon Press).* The Institutional Animal Care and Use Committee of Southern Medical University approved all the protocols of zebrafish operations.

**Figure 1:**
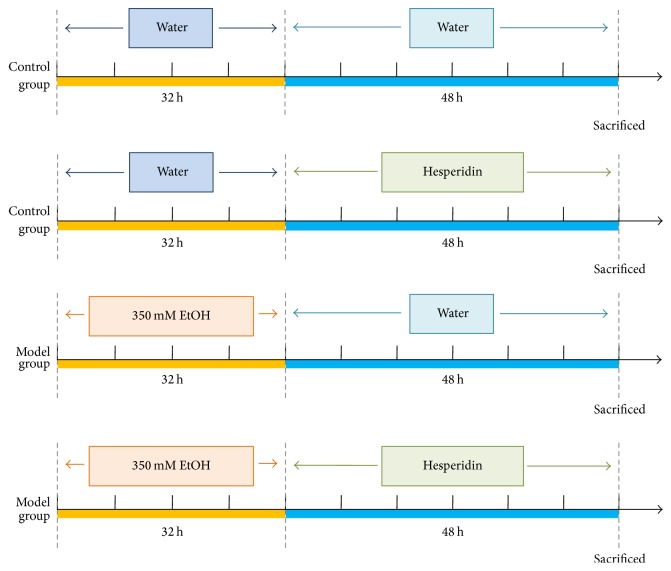
Experimental plan for zebrafish.

96–98 hours after fertilization (hpf) zebrafish larvae were first randomly divided into two groups, a control group treated with system water (water out of the water system of culture facility for zebrafish) only and a model group exposed to 350 mM ethanol for 32 h [8]. Subsequently, the control larvae were randomly divided into two groups (*n* = 40 in each group): a control group (treated with system water) and a hesperidin control group (treated with 25 *μ*g/mL hesperidin). Simultaneously, the model larvae were randomly assigned into several groups as followed equally (*n* = 40 in each group): a model group (treated with system water) and 3 hesperidin treated groups (25 *μ*g/mL, 12.5 *μ*g/mL, and 6.25 *μ*g/mL). Hesperidin monomer was dissolved in 0.1% DMSO (diluted in system water). After being incubated for 48 h, larvae were collected for further detection. The experimental plan for zebrafish is shown in Figure 1.

### 2.2. Oil Red O Staining

Zebrafish larvae of each group were collected and fixed with 4% paraformaldehyde (PFA) overnight at 4°C, washed 3 times with phosphate-buffered saline (PBS), and infiltrated sequentially with 20%, 40%, 80%, and 100% propylene glycol (Sigma, USA) at room temperature for 15 min, respectively. Subsequently, the larvae were stained with 0.5% Oil Red O (Sigma, USA) in 100% propylene glycol in the dark for 1 h at 65°C. Then the samples were destained by soak sequentially in 100%, 80%, 40%, and 20% propylene glycol for 30 min, respectively, and washed 3 times with PBS, followed by storing in 70% glycerol (Sigma, USA) [9]. The hepatic morphology and lipid droplets in liver were observed and imaged with microscope (Olympus szx10, Tokyo, Japan). In this study, staining shade and liver size were quantized into gray values by Image J software in order to reflect the degree of hepatic steatosis.

### 2.3. Nile Red Staining

The procedures were performed as previously described [10, 11]. Zebrafish larvae were fixed with 4% PFA as described previously and incubated in citric acid with 0.1% Triton (Sigma, USA) for 2 hours at 65°C after being washed with PBS 3 times. DAPI (Solarbio Life Science, China) was counterstained in the dark for 10 minutes at room temperature to stain the nuclei. Subsequently Nile Red dye (0.5 *μ*g/mL in acetone, Sigma, USA) was used to stain the lipid droplets in liver, incubated in the dark for 50 minutes at room temperature, and washed 3 times with PBS. The stained larvae were imaged with Confocal Laser Scanning Microscope (Nikon C2plus, Tokyo, Japan).

### 2.4. Histologic Analysis

Zebrafish larvae were fixed with 4% PFA overnight, penetrated with ethanol and xylene respectively, embedded in paraffin, cut into 4 *μ*m thick sections, stained with H&E, and observed with microscope (Nikon Eclipse Ni-U, Tokyo, Japan).

### 2.5. Quantitative Real-Time PCR

The procedure was performed according to the previous study [12]. Total RNA was extracted from 10 zebrafish larvae using Trizol reagent (Invitrogen, USA) following the standard procedures and subsequently reverse-transcribed with qScript cDNA using PrimeScript™ RT-PCR Kit (Takara). qPCR was carried out on Light Cycler 96 (Roche, Switzerland) using a SYBR Green kit (Takara Biotechnology, Inc.). The detailed protocol outlined by the manufacturer's instructions was followed. The levels of target genes were calculated by the comparative CT method and normalized to the reference gene* rpp0* (ribosomal protein P0). Primers for each gene are listed in Table 1.

### 2.6. Statistical Analysis

All data are presented as mean ± standard error of the mean (SEM). Statistical analysis was carried by SPSS (version 20.0). Statistical differences were evaluated by Student's *t*-test and one-way ANOVA test. Value of *P* < 0.05 was considered to be statistically significant. GraphPad Prism 5 software was used to plot graph.

## 3. Results

### 3.1. Alcoholic Fatty Liver Model Was Established in Zebrafish Larvae

96–98 hpf zebrafish larvae were chosen to be exposed to ethanol during a window, which was the stage from the formation of liver to the full utilization of yolk (5.5–6 dpf). During this period the metabolic effects of fasting could be avoided [13]. The acute alcoholic exposure time of zebrafish larvae was set to 32 hours, which is used to distinguish it from chronic exposure in alcoholics.

**Figure 2:**
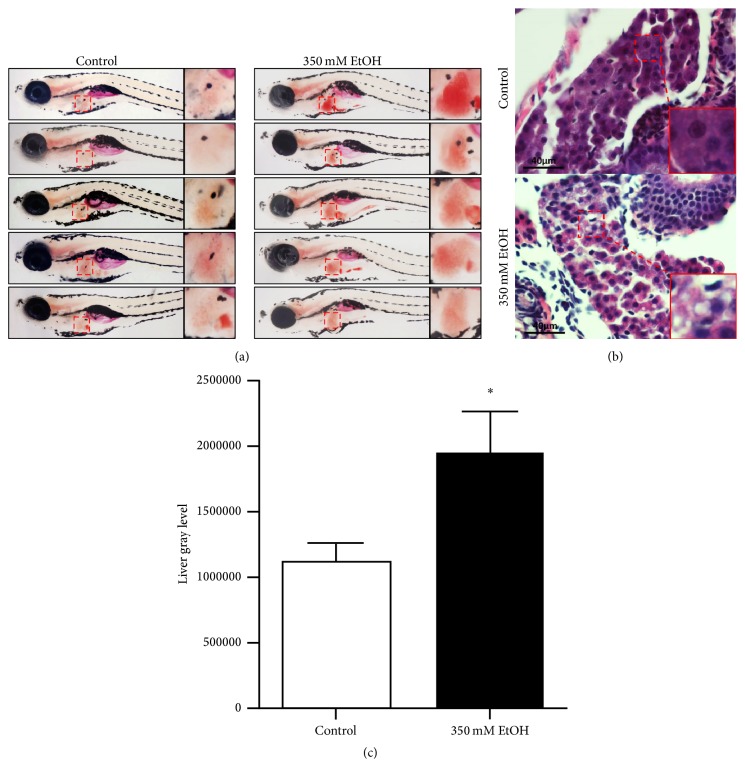
*Alcoholic fatty liver model was established in zebrafish larvae*. (a) Oil Red O staining for whole body of zebrafish larvae. (b) H&E staining for liver sections of zebrafish larvae. (c) Quantitative analysis for the results of Oil Red O staining (*n* = 20/group, three experiments). The data are presented as the means ± SEM (^*∗*^*P* < 0.05 versus control group).

Taking previous studies into account, we discovered that morphological phenotypes, hepatomegaly, and behavioral abnormalities occurred in most of the larvae after having been treated with 350 mM ethanol for 32 hours [14, 15]. Histologic examinations of liver stained with H&E and Oil Red O revealed that severe lipid deposited in the liver tissues after 32 hours of exposure to 350 mM ethanol (Figures 2(a) and 2(b)). Furthermore, we discovered that 350 mM ethanol could lead to hepatic steatosis in zebrafish larvae after 32 hours of treatment, by quantification of Oil Red O staining in the liver, performed by Image J software (Figure 2(c)).

### 3.2. Hesperidin Reduced Hepatic Steatosis in Zebrafish Larvae Induced by Alcohol

**Figure 3:**
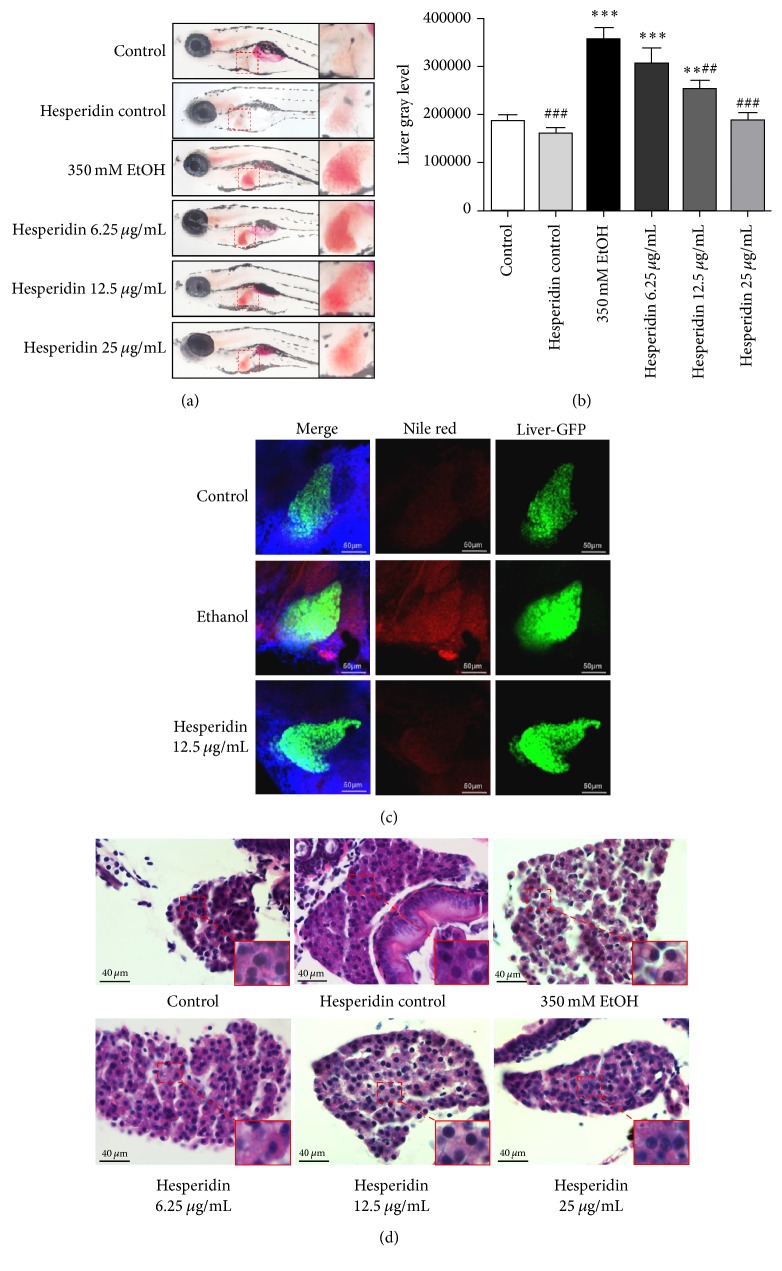
*Hesperidin reduced hepatic steatosis in zebrafish larvae induced by alcohol*. (a) Oil Red O staining for whole body of zebrafish larvae. (b) Quantitative analysis for the results of Oil Red O staining (*n* = 20/group, three experiments). (c) Nile Red staining for intracellular lipid droplets in liver tissues of zebrafish larvae. (d) H&E staining for liver sections of zebrafish larvae. The data are presented as the means ± SEM (^*∗*^*P* < 0.05 versus control group; ^#^*P* < 0.05 versus 350 mM EtOH group).

As descried above, there existed severe lipid deposits in the liver tissues in larvae after alcoholic exposure. However, it was interesting that hesperidin could dose-dependently alleviate hepatic steatosis in larvae induced by alcohol (Figure 3(a)). The development of hepatic steatosis was quantified into gray level according to the results of Oil Red O staining by Image J software. The assessment of gray level further showed that hesperidin could reduce the development of hepatic steatosis with a dose-dependent correlation. The dose of 12.5 *μ*g/mL and 25 *μ*g/mL almost reversed the alcoholic lipid deposition in larvae (Figure 3(b)). On the other hand, using the Nile Red staining, a selective fluorescent dye for intracellular lipid droplets, we investigated whether hesperidin had a protective effect on liver of* Tg (lfabp10α:eGFP)* larvae after alcoholic exposure. Consistent with the results of Oil Red O staining, hesperidin (12.5 *μ*g/mL, 48 hours) significantly alleviated hepatic lipid droplets induced by alcohol in larvae (Figure 3(c)). Furthermore, paraffin sections of larvae stained with H&E also confirmed the liver pathological changes consistently (Figure 3(d)). Additionally, Oil Red O staining and H&E staining showed that hesperidin does not have any substantial effects on livers of control zebrafish (Figures 3(a), 3(b), and 3(d)).

### 3.3. Hesperidin Improved Alcohol Metabolism in Zebrafish Larvae

We further investigated the effects of hesperidin on alcohol metabolism. Cytochrome P450 family 2 subfamily E member 1* (cyp2e1)*, a crucial enzyme in regulation of oxidative stress response in alcohol metabolism process, is considered to be responsible for alcoholic liver injury in mammals. Cytochrome P450 family 2 subfamily Y polypeptide 3* (cyp2y3)*, a gene homolog of cyp2e1, is essential for alcohol metabolism in liver of zebrafish [13]. Liver injury is dramatically increased due to the increase of* cyp2y3*, which could speed up the rate of alcohol metabolism and accumulation of acetaldehyde [13]. As showed in Table 2, the expression of* cyp2y3* mRNA was significantly increased compared with the control larvae. Interestingly, hesperidin intervention normalized the level of* cyp2y3* mRNA in larvae. Moreover, a similar change of the expression of cytochrome P450 family 3 subfamily A polypeptide 65* (cyp3a65)* occurred, which is a homo gene of cytochrome P450 family 3 subfamily A* (cyp3a)* primarily in the liver and crucial to the metabolisms of both endogenous and exogenous substances [16]. These findings indicated that hesperidin might improve alcohol metabolism and reduce the accumulation of toxic substances in zebrafish larvae after exposure to ethanol.

### 3.4. Hesperidin Protected Zebrafish Larvae against Alcoholic Injury through Improving Lipid Metabolism

We further investigated some lipid metabolism related genes (*hmgcra*,* hmgcrb*,* hmgcs*,* fasn*, and* fads2*), which were related to cholesterol synthesis, fatty acid synthase, desaturase, and mitochondrial enzyme, in order to confirm whether hesperidin could protect against hepatic steatosis by reduction of lipid metabolism and improvement of lipid homeostasis [17–20]. The results of qPCR showed that the expressions of* hmgcra*,* hmgcrb*,* hmgcs*,* fasn*, and* fads2 *mRNAs were significantly increased in larvae after treatment with alcohol. However, the intervention of hesperidin induced the levels of these mRNAs above to reversion (Table 3).

### 3.5. Hesperidin Reduced Endoplasmic Reticulum Stress and DNA Damage Induced by Alcohol in Zebrafish Larvae

Endoplasmic reticulum stress and DNA damage play key roles in various kinds of pathological liver damage induced by alcohol [21, 22]. We investigated the levels of mRNAs, DNA damage inducible transcript 3* (chop)*, growth arrest, and DNA damage-inducible, *α*, a* (gadd45αa)* and endoplasmic reticulum degradation-enhancing *α*-mannosidase-like protein 1* (edem1)*, which were related to endoplasmic reticulum stress and DNA damage [22–24]. The results of mRNAs levels also confirmed that hesperidin normalized the increased expressions of* chop*,* gadd45αa*, and* edem1* induced by alcohol (Table 4). Collectively, these evidences indicated that hesperidin suppressed endoplasmic reticulum stress and DNA damage.

## 4. Discussion

Hepatic steatosis, the earliest manifestation of alcoholism, can develop into some severe liver diseases [2]. Hepatocytes are susceptible to damage due to chronic hepatic steatosis, which is generally the early stage of steatohepatitis and cirrhosis [25]. Thus, further liver damage induced by alcohol can be prevented through the blockade of lipid accumulation. Moreover, it is reported that hesperidin in vivo can improve certain aspects of lipid homeostasis and reduce inflammation of adipose tissue [26]. However, there is no study about the effects of hesperidin on alcohol and metabolic abnormalities. To our knowledge, it is the first time that we investigated the effects of hesperidin on regulating alcohol metabolism, pathology, endoplasmic reticulum stress, and DNA damage in ALD on zebrafish. In this study, according to previous findings [14, 15], we successfully established an ALD zebrafish model by exposing zebrafish larvae to 350 mM ethanol for 32 hours. In addition, we discovered that the intervention of hesperidin could inhibit hepatic steatosis and endoplasmic reticulum stress of hepatocytes induced by acute alcoholic exposure.

The establishment of ALD zebrafish larvae is easy to operate and less time-consuming. Given that there exists difficulties of gaining liver tissues and blood from zebrafish larvae, we are not able to investigate the expressions of mRNAs and proteins of liver tissues or the serum levels of biochemical markers of liver injury directly. However, zebrafish larvae show more advantages on short growth cycle and transparent body, so we can obtain quantities of larvae in a short time and it is easier to get observation of the overall staining.

We discovered hesperidin protected against hepatic steatosis in zebrafish larvae after alcoholic exposure for the first time in this present study. Larvae stained with H&E and Oil Red O indicated that hesperidin could attenuate alcohol-induced hepatic steatosis and its therapeutic effect was dose-dependent. Moreover, the best and lowest treatment concentration is 12.5 *μ*g/mL. Now that the antisteatosis effect of hesperidin was confirmed, we then investigated the possible effects of hesperidin against cell death and damage induced by alcohol. In addition, both* chop* and* gadd45αa* can inhibit cell growth while increasing cell damage [22, 23]. Transcription of lipid metabolism can be regulated by* chop*, the upregulation of which can lead to abnormal lipid metabolism in the liver [27]. Moreover,* chop* is considered as a specific transcription factor of endoplasmic reticulum stress [22]. In another aspect,* edem1*, a gene essential for the unfolded protein response, was upregulated markedly with endoplasmic reticulum stress unbalance [24]. After exposure to alcohol, the expressions of* chop, gadd45αa*, and* edem1* were significantly increased in larvae, which indicated that the larvae were going through severe endoplasmic reticulum stress and DNA damage during that period. To the contrary, downregulation of chop, gadd45*α*a, and edem1 were induced in larvae after being treated with hesperidin. Collectively, we summed up that hesperidin could inhibit steatosis and damage of liver in zebrafish larvae after alcoholic exposure.

HMG-CoA reductases are key enzymes in lipid metabolism, including HMG Coenzyme A reductase a* (hmgcra),* HMG Coenzyme A reductase b* (hmgcrb)*, and 3-hydroxy-3-methylglutaryl-CoA synthase* (hmgcs)*, mainly regulating genes related to cholesterol synthesis [14, 17, 28]. Besides, synthesis and desaturation of fatty acid can be regulated by fatty acid synthase* (fasn)* [19]. Fatty acid desaturase 2* (fads2)*, a gene related to dyslipidemia, primarily participates in metabolism of unsaturated fatty acids, affecting the concentrations of total cholesterol, low density lipoprotein cholesterol, high lipoprotein cholesterol, and triglyceride [18]. In our study, the expressions of* hmgcra*,* hmgcrb*,* hmgcs*,* fasn,* and* fads2* genes related to lipid metabolism were significantly increased in larvae after alcoholic exposure, which indicated that treatment with alcohol could cause lipid metabolism disorders in zebrafish larvae. However, hesperidin markedly ameliorated lipid metabolism through mediating the expressions of these genes above.

**Figure 4:**
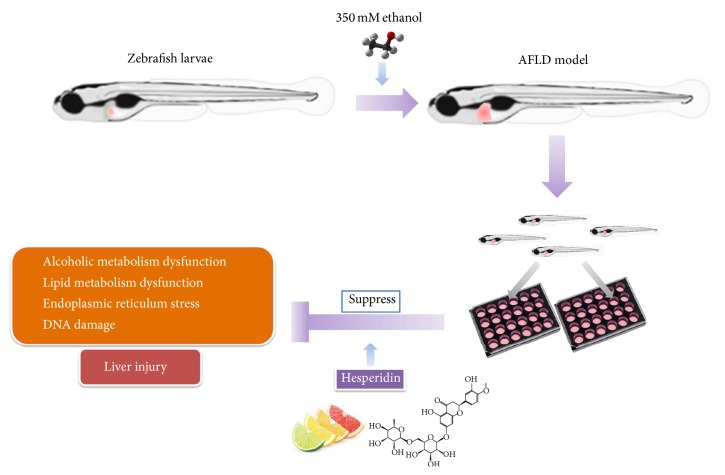
A model depicting the protective role of hesperidin in zebrafish larvae during acute alcoholic injury.

In another aspect,* cyp2y3* and* cyp3a65*, homologous genes of cytochrome P450 CYP2* (cyp2)* and* cyp3a*, are essential for alcoholic metabolism mainly in liver of zebrafish. The closest homolog to* cyp2e1* in zebrafish is* cyp2y3*, which has a protein similarity of 43% [13]. Alcohol metabolism and oxidative stress can be decreased by blocking* cyp2* homologous genes. In addition,* cyp3a65* is crucial to metabolism of both endogenous and exogenous substances [16]. Interestingly, we found that the treatment of hesperidin could reduce the levels of* cyp2y3* and* cyp3a65 *in larvae, which were upregulated by alcoholic exposure previously. The underlying mechanism of the therapeutical effect of hesperidin was likely to be related to the improvement of alcoholic metabolism and reduction of toxic substances. Taking all these evidences above, we discovered that alcohol-induced liver injury of zebrafish larvae was mainly caused by dysbolisms of lipid and alcohol. However, these dysbolisms could be improved by hesperidin, which resisted alcohol-induced steatosis and injury therefore. Finally, we summarized the protective effects of hesperidin in zebrafish larvae during acute alcoholic injury as showed in Figure 4.

In conclusion, we revealed that hesperidin inhibited hepatic steatosis and injury in zebrafish induced by alcohol, by ameliorating cell damage and regulating metabolism of alcohol and lipid. However, the pathways of effects of hesperidin on reducing cell damage and lipid metabolism still need further exploration. Hesperidin is abundant in citrus fruits and grape fruit [26], which indicates that hesperidin easily accumulates in the plasma and is available in vivo when humans intake hesperidin-containing food regularly. Thus, whether hesperidin is suitable for prevention of ALD and lipid metabolism syndrome needs further preclinical investigation.

## Figures and Tables

**Table 1:** Primers used to quantify mRNA levels.

Gene	FP sequence (5′-3′)	RP sequence (5′-3′)
*cyp2y3*	tattcccatgctgcactctg	aggagcgtttacctgcagaa
*cyp3a65*	aaaccctgatgagcatggac	caagtctttggggatgagga
*hmgcra*	ctgaggctctggtggacgtg	gatagcagctacgatgttggcg
*hmgcrb*	cctgttagccgtcagtgga	tctttgaccactcgtgccg
*hmgcs *	ctcactcgtgtggacgagaa	gatacggggcatcttcttga
*fasn *	gagaaagcttgccaaacagg	gagggtcttgcaggagacag
*fads2 *	tcatcgtcgctgttattctgg	tgaagatgttgggtttagcgtg
*chop *	aggaaagtgcaggagctgac	ctccacaagaagaatttcctcc
*gadd45αa *	tggctttgtttgtgggactt	tggaaaacagtccactgaga
*edem1*	gacagcagaaaccctcaagc	catggccctcatcttgactt
*rpp0 *	ctgaacatctcgcccttctc	tagccgatctgcagacacac

**Table 2:** Hesperidin treatment improved alcohol metabolism in zebrafish larvae.

mRNA level (versus *rpp0*)	Group		
	Control	350 mM EtOH	Hesperidin (12.5 *μ*g/mL)
*cyp2y3*	1.659*e* − 4 ± 3.574*e* − 5	3.04*e* − 4 ± 3.018*e* − 5^*∗*^	1.677*e* − 4 ± 3.799*e* − 5^#^
*cyp3a65*	1.565*e* − 2 ± 5.0*e* − 5	1.77*e* − 2 ± 4.0*e* − 4^*∗*^	1.135*e* − 2 ± 2.5*e* − 4^*∗∗*##^

*n* = 20/group, three experiments; the data are presented as the means ± SEM (^*∗*^*P* < 0.05 versus control group; ^#^*P* < 0.05 versus 350 mM EtOH group).

**Table 3:** Hesperidin treatment improved lipid metabolism in zebrafish larvae against alcoholic injury.

mRNA level (versus *rpp0*)	Group		
	Control	350 mM EtOH	Hesperidin (12.5 *μ*g/mL)
*hmgcra*	1.762*e* − 4 ± 8.408*e* − 6	5.378*e* − 4 ± 1.006*e* − 4^*∗∗∗*^	3.048*e* − 4 ± 2.663*e* − 5^#^
*hmgcrb*	3.442*e* − 4 ± 7.15*e* − 6	6.391*e* − 4 ± 1.011*e* − 4^*∗*^	2.564*e* − 4 ± 3.55*e* − 6^#^
*hmgcs*	1.575*e* − 4 ± 5.5*e* − 6	1.87*e* − 4 ± 5.0*e* − 6	1.305*e* − 4 ± 1.15*e* − 5^#^
*fasn*	6.41*e* − 4 ± 3.1*e* − 5	8.5*e* − 4 ± 6.0*e* − 6^*∗∗*^	2.32*e* − 4 ± 1.0*e* − 5^*∗∗*###^
*fads2*	1.338*e* − 4 ± 3.525*e* − 5	4.79*e* − 4 ± 4.8*e* − 5^*∗*^	0.462*e* − 4 ± 1.04*e* − 5^##^

*n* = 20/group, three experiments; the data are presented as the means ± SEM (^*∗*^*P* < 0.05 versus control group; ^#^*P* < 0.05 versus 350 mM EtOH group).

**Table 4:** Hesperidin attenuates endoplasmic reticulum stress and DNA damage in zebrafish larvae with alcoholic injury.

mRNA level (versus *rpp0*)	Group		
	Control	350 mM EtOH	Hesperidin (12.5 *μ*g/mL)
*chop*	7.459*e* − 3 ± 2.27*e* − 3	13.34*e* − 3 ± 5.576*e* − 4^*∗*^	7.778*e* − 3 ± 1.029*e* − 3^#^
*gadd45αa*	8.41*e* − 4 ± 3*e* − 6	12.65*e* − 4 ± 4.5*e* − 5^*∗∗*^	8.93*e* − 4 ± 2.8*e* − 5^##^
*edem1*	1.739*e* − 4 ± 4.2*e* − 6	3.007*e* − 4 ± 8.3*e* − 6^*∗∗*^	1.427*e* − 4 ± 1.675*e* − 5^##^

*n* = 20/group, three experiments; the data are presented as the means ± SEM (^*∗*^*P* < 0.05 versus control group; ^#^*P* < 0.05 versus 350 mM EtOH group).

## Abbreviations

ALD: Alcoholic liver disease
PBS: Phosphate-buffered saline
PFA: Paraformaldehyde
H&E: Hematoxylin and eosin
qPCR: Real-time quantitative PCR
hpf: Hours after fertilization
dpf: Days after fertilization
rpp0: Ribosomal protein P0
cyp2e1: Cytochrome P450 family 2 subfamily E member 1
cyp2y3: Cytochrome P450, family 2, subfamily Y, polypeptide 3
cyp3a65: Cytochrome P450, family 3, subfamily A, polypeptide 65
cyp3a: Cytochrome P450, family 3, subfamily A
chop: DNA damage inducible transcript 3
gadd45*α*a: Growth arrest and DNA damage-inducible, alpha, a
hmgcra: HMG Coenzyme A reductase a
hmgcrb: HMG Coenzyme A reductase b
fasn: Fatty acid synthase
fads2: Fatty acid desaturase 2
fabp10*α*: Fatty acid binding protein 10a
edem1: Endoplasmic reticulum degradation-enhancing *α*-mannosidase-like protein 1.
